# Exploring mental health literacy among youths with background as asylum-seekers and refugees: a systematic review

**DOI:** 10.3389/fpsyt.2025.1538946

**Published:** 2025-04-08

**Authors:** Eli Sandsgård-Hilmarsen, Eline Ree, Anita Salamonsen, Petter Viksveen

**Affiliations:** Department of Quality and Health Technology, Faculty of Health Sciences, University of Stavanger, Stavanger, Norway

**Keywords:** asylum-seekers, mental health, mental health literacy, mental health problems, mental health services, refugees, systematic review, youths

## Abstract

The purpose of this study was to explore mental health literacy among youths with a background as asylum-seekers and refugees including the role of mental health literacy as a barrier to their service use. A systematic literature study was conducted to gain an overview of mental health literacy in youths with a background as asylum-seekers and refugees. The databases MEDLINE, PubMed, CINAHL, PsycINFO and Web of Science were used to identify relevant research. The PRISMA statement was used to report on the literature search, and a thematic synthesis was used to analyze the data from the included studies. Eleven studies reporting qualitative data were included. The understanding of mental health varied. Some youths understood mental health and mental health problems as connected to body and mind, whereas others linked it to their life situation or attributed it to supernatural or religious causes. Help-seeking behavior also varied, with youths seeking support in ways they found helpful, such as through prayer, or talking to an Imam or priest, or confiding in friends and family. Professionals were often perceived as unhelpful, partly due to lack of cultural competency. Youths expressed mistrust of professionals, feeling that they focused too much on their past rather than addressing their present and future concerns. Moreover, mental health stigma was a significant barrier to seeking help. Different understandings of mental health among youths with a background as asylum-seekers and refugees influence their help-seeking behaviors. The current conceptual framework of mental health literacy does not address cultural and contextual factors. Mental health literacy should be further developed as a concept to encompass diverse perspectives. Professionals should be trained with a more holistic approach that considers cultural factors and user experiences, guiding the development of mental health literacy programs and services.

## 1 Introduction

There were 110 million forcibly displaced people worldwide at the end of June 2023, of which 36.5 million were refugees, and 6.1 million asylum-seekers (1). The Office of the United Nations High Commissioner for Refugees (2) has estimated that around 40% of refugees are children under the age of 18. Refugees are persons who flee their country for reasons related to fear of persecution due to ethnicity, religious affiliation, nationality, political opinion, or membership of a particular community; and who are unable to return to their home country (2). Asylum-seekers are persons who flee to another country and apply for asylum status and protection as refugees for political, ethnic or religious reasons. Asylum-seekers are not considered refugees but may be granted residence on humanitarian grounds (3). The terms asylum-seekers and refugees are not to be confused with the term migrants, who have voluntarily chosen to leave their country and who may return home without risking their life and safety (2). The term refugee will for the most part be used in the article to report on both asylum-seekers and refugees.

The migration process, which in this context is referred to as the transition of refugees from their country of origin to the country of destination, includes settling in a new and unfamiliar country and adapting to a new culture, as well as managing any after-effects of traumatic pre-migration and transit experiences. Each phase can impact health, in particular mental health (4). Post-migration stressors may include financial challenges, language barriers, settling in unfamiliar physical and cultural environments, experiences of discrimination, loss of family, and challenges associated with cultural identity, social isolation and extensive asylum-seeking processes. Post-migration stressors may be just as significant as pre-migration traumatic experiences (5).

The prevalence of mental health problems has been found to be higher among refugee youths than among the majority youth population (6). The United Nations (UN) categorizes youths as the age range of 15 to 24 (7). Internationally, many youth mental health services span the age range 16 to 25 (8), and youths have the right to consent to their treatment at the age of 16 (9). A systematic review and meta-analysis from 2020 found that post-traumatic stress disorder (PTSD), anxiety and depression are the most common mental health problems, with prevalence rates of 23%, 16% and 14%, respectively (6). The prevalence of PTSD, anxiety and depression is higher within the two first years of displacement (6). It seems that unaccompanied minors are more vulnerable with a higher prevalence of mental health problems, compared to youths who are accompanied by adults (10). Even though the prevalence of mental health problems is high among refugee youths, few seek professional help for their problems (11). Low mental health literacy has been found to be one of the reasons for this among young people (12), and refugee youths in particular (13). To date, no definition describes how MHL should be understood within the youth population.

Mental health literacy (MHL) was first defined in 1997 by Jorm et al. (14) as *“knowledge and beliefs about mental disorders which aid their recognition, management or prevention”* (p.182). The term “mental health problems” will from here on refer to self-reported mental health problems, as well as to diagnosed or undiagnosed mental illness or disorders, unless a specific distinction is necessary for the context.

The concept of mental health literacy has been subject to criticism over the last years. For example, Chambers et al. (15) have pointed out that the definition of mental health literacy at the time was restrictive, focusing primarily on identification of mental disorders. Nevertheless, the MHL is still extensively used as a concept internationally and instruments have been developed to measure levels of MHL in individuals (16, 17).

Many scholars refer to Jorm’s definition of MHL (14, 18), but over the past decade, several attempts have been made to establish a broader definition. MHL now also includes how to obtain a positive mental health; how to decrease stigma related to mental health problems; and how to enhance help-seeking efficacy (19). Mental health stigma encompasses both public stigma and self-stigma. Public stigma refers to societal prejudices and negative attitudes towards individuals with mental health problems, while self-stigma involves the internalization of public stigma which in turn can lead to poor self-esteem (20, 21). Stigma may be a significant barrier to MHL, as it is associated with lack of knowledge about mental health and mental health problems, preventing people from recognizing symptoms and seeking help (11, 13). There is no consensus among scholars on the specific components that should be included in the definition of MHL (17).

Furnham and Swami suggested in their review (22) that MHL is generally lower in lay people compared to mental health professionals, though it has increased in some industrialized countries, where causal explanations among lay people align with those of professionals. However, MHL remains low in low- and middle-income countries (LMICs) particularly in regions where many refugee populations originate, including the Middle East, Africa, Asia, and South America. High MHL is characterized by recognizing mental health problems as biologically driven, using established diagnostic terms, and seeking help from professionals. Members of the general population more commonly use psychosocial over biological models; fail to use correct diagnostic terms; and seek help form peers, rather than from professionals (22). Factors such as lower sociodemographic status, higher age, lower education levels, strong religious beliefs, and limited experience with mental health problems in oneself or in close others are associated with poorer MHL (22). Lack of symptom recognition is a barrier to seeking help (22).

Spiker and Hammer (23) suggest to further develop MHL theory. Initially, this requires a need for an agreement about its definition. A recent scoping review and Delphi panel by Soria-Martínes et al. (24) identified the following components in a conceptual framework for mental health literacy:

- Knowledge and skills that enhance understanding of mental health.
- Knowledge and skills that enhance understanding of mental disorders.
- Knowledge and skills that enhance understanding of treatments for mental disorders.
- Knowledge and skills that improve access to and management of information related to mental health.
- Attitudes and stigmatizing beliefs towards mental health problems.

According to this definition (24), a person with higher mental health literacy should possess knowledge about the causes of mental health problems, be able to recognize their symptoms and severity, and know about available treatment options. Furthermore, the person should be able to identify where and when to seek help, access and apply relevant information, possess skills necessary for self-help and self-management of mental health problems, as well as to be able to support others and understand how to achieve and maintain a good mental health (24). Moreover, the authors suggest that MHL encompasses the ability to understand biological, psychological, and social aspects of mental health, as well as how socio-demographic factors can influence it. Stigma and negative attitudes towards mental health are associated with low MHL, whereas awareness of these issues is associated with higher levels of MHL (24). Culture, age and gender can affect the level of MHL (22). For example, persons within different cultures have varying understandings of risk factors and causes of mental health problems (24). Some findings suggest that MHL influences help-seeking behavior, and it is associated with self-help and coping strategies (25).

Culture and religion play an important role within groups of culturally and linguistically diverse youths, and the transition to new social environments with different norms can negatively impact their mental health (25). A culturally diverse population requires culturally sensitive care to feel comfortable, respected and to build trust in the healthcare services in general and healthcare professionals in particular. Conversely, lack of cultural sensitivity may lead to dissatisfaction with services, misunderstandings and poorer health outcomes (26). Even though the concept of MHL has been subject to criticism, it is still extensively used and specific tools are used to measure MHL (26). Therefore, there is a need for a review of the existing literature that explores MHL among youths with a refugee background, to better understand their specific needs for support and mental health services. Hence, the aim of this study was not to measure the levels of mental health literacy among youths with refugee backgrounds, but to explore their understanding of mental health and their use of mental health services, as well as to determine how and to what extent the existing definition of MHL in their opinion is appropriate or should be adapted.

The purpose of this systematic review was to gain insight into the existing research literature reporting on MHL among asylum-seeker and refugee youths aged 16 to 24 years. MHL is in this study based on the definition by Soria-Martínes et al. (24).

## 2 Methods

### 2.1 Study design

We conducted a systematic review, applying the Preferred Reporting Items for Systematic reviews and Meta-Analyses (PRISMA) check-list (27). A systematic review protocol was registered in the International Prospective Register of Ongoing Systematic Reviews (PROSPERO ID: CRD42024481311).

### 2.2 Inclusion and exclusion criteria

The inclusion criteria were research articles reporting on youths in the age range from 16 to 24 years with a background as asylum-seekers or refugees. There is variation in how different institutions and organizations internationally define youth. While the youngest youth are typically considered to be 15 or 16 years old, the upper age limit varies more extensively. For example, the UN habitat definition includes individuals up to the age of 32 years, and the African Youth Charter includes individuals up to 35 years old (28). However, there are several arguments for setting the upper age range to 25, as used in the UN guidelines for youth development (28). Although we acknowledge that this age limitation may exclude potentially relevant articles focusing on younger or older youths, the chosen range is broadly accepted and justified by its alignment with developmental, mental health services, and policy frameworks. This focus ensures that the study’s findings are both meaningful and applicable to the target population. A minimum of 50% of the participants had to be in the age range and have a background as asylum-seekers or refugees. Moreover, the articles had to report on at least one component of MHL in line with Soria-Martínez et al.’s definition (24). All types of peer reviewed quantitative, qualitative, and mixed methods studies in English were included, except intervention studies which aimed to improve mental health literacy, posters, conference abstracts and literature reviews. No time limitation was set. General health literacy, i.e. health literacy not specific to mental health, was an exclusion criterion.

### 2.3 Search strategy

A preliminary search was conducted in EMBASE in November 2023. The scope was to identify additional potentially relevant search terms, including MeSH terms, as well as to test different search strategies. The inclusion of “mental health” in addition to “mental health literacy” as search terms, resulted in identification of several titles which were not relevant to the systematic review. However, the term “mental health” was retained as some relevant articles would otherwise have been left out. The search strategy was discussed, revised and finalized within the research team. A university librarian was consulted during the planning process.

The literature search was conducted independently by two researchers (ESH and PV) from December 2023 to January 2024, and was performed using the following databases: EMBASE, PsycINFO, CINAHL, Web of Science, MEDLINE, and PubMed. Additionally, the reference lists of all included articles were checked for identification of additional titles. The search strategy was customized to the individual databases. The search strategy included both text words and where available, MeSH terms. The Boolean operator “OR” was used to expand searches, whereas the operator “AND” was used to limit searches. Moreover, the asterisk * was used as a wildcard to include variations of a root word. An example of the search strategy: (Youth* OR Young OR young adult* OR young people) AND (refugee* OR asylum-seeker* OR asylum OR fugitive* OR displaced people) AND (“mental health literacy” OR “mental health knowledge” OR “mental health belie*” OR “mental health awareness” OR “attitudes towards mental illness” OR “attitudes to mental health” OR “mental health stigma” OR “mental illness stigma” OR “mental health help-seeking behavior” OR “mental health”). A more complete description of the search strategy can be found in **Supplementary File 1**.

The screening process was performed in two stages. The first stage consisted of screening titles and abstracts, using the Rayyan screening tool (29). In the second stage, the remaining full text articles were considered for inclusion. Discrepancies between the two researchers’ findings were discussed and consensus on inclusion and exclusion of articles was reached.

### 2.4 Quality assessment

The methodological quality of the included articles was assessed using the Standards for Reporting Qualitative Research (SRQR) guidelines, as only qualitative data in the included studies could be used for the data extraction and analysis in this review (30). The SRQR is an assessment tool which contains 21 questions regarding six different aspects of a study. Each item was assessed with a score of Yes (1), Partly (0.5), No or Unclear (0) (31). Quality assessment was performed by one researcher (ESH) and checked by another (PV). Agreement on quality assessment was reached for all included articles.

### 2.5 Data extraction

The data extraction tool was based on recommendations provided by Cherry et al. (32). These recommendations facilitated decisions concerning the study characteristics that were to be extracted (32). The inclusion and exclusion criteria, as well as the five components included in the MHL definition provided by Soria-Martínez et al. (24), were used to extract participants’ characteristics and results of individual studies.

Data was extracted from the results section in the included studies. Only the results of youths’ perspectives were extracted from studies which also included other stakeholders such as healthcare professionals. Data was extracted by one researcher (ESH) and checked by another (PV). Agreement on data extraction was reached for all included articles.

### 2.6 Data analysis

Data analysis was conducted using thematic synthesis (33). Thematic synthesis is based on different qualitative methods, such as thematic analysis and meta-ethnography. It is used to identify key concepts from primary studies, transfer these across the studies and build a general interpretation as known in “meta-ethnography” and in the process of thematic analysis (33). Based on this approach (33), we analyzed the data through a three-step process where the data material was first coded line by line, followed by development of descriptive themes, and finally development of analytical themes. Each stage is described in further detail below. The approach was chosen as it can be used both inductively and deductively (34). In addition to providing a descriptive presentation of the results, it allows for interpretation of the data (33). Even though the current review was based on the conceptual framework of MHL as defined by Soria-Martínes et al. (24), the aim was not to measure the level of MHL, but to explore the participants’ perspectives. Therefore, the process focused also on the participants’ perspectives on mental health and help-seeking behaviors. This is important for the further development of the conceptual MHL framework. We decided to analyze the data using standard text software (Word for Microsoft 365 MSO Version 2408) as this provided flexibility in formatting and organizing data. Moreover, the approach was feasible given the limited amount of data. This allowed us to go back and forth in the data, adapting the codes as needed to retain the meaning.

#### 2.6.1 Coding

The first step consisted of reading and becoming familiar with the data. The first author (ESH) coded the data line by line (in Word). The codes were discussed with the last author (PV), revised by the first author, and checked by the last author. Data extracts and accompanying codes were then sent to the other two authors (AS and ER) who provided their input. Consensus was reached for all codes, which were kept as close to the original data material as possible in order to preserve their meaning and content (34).

#### 2.6.2 Development of descriptive themes

Codes were first categorized into the different components of MHL (in Excel) (24). This resulted in development of preliminary themes. Some themes overlapped and others were later found to contain insufficient data material to stand on their own. Moreover, there was a risk of overlooking meanings embedded in the data material when attempting to categorize the themes according to the MHL conceptual framework. Descriptive themes and subthemes were therefore reorganized by comparing codes, collapsing similar codes, and regrouping them into themes that better described the meaning of the content (33). The themes and subthemes were revised several times until codes and themes did not overlap. Reflecting on our own preconceptions and trying to see the content from an as neutral perspective as possible, was a part of the process. Throughout the process, the codes in the different themes were checked repeatedly against the data material to try to ensure all relevant content was preserved. We aimed for theme titles to be descriptive of the content of the data material. This process was led by the first author and discussed several times with all authors in order to reach consensus on descriptive themes.

#### 2.6.3 Development of analytical themes

The purpose of the analytical themes was to go beyond the descriptive themes, which means that the analysis was not only based on the raw data but included connections and patterns which were not discovered at first glance. While the descriptive themes are typically close to the data material, the analytical themes provide a deeper understanding of the descriptive themes, equivalent to a third-order interpretation in meta-ethnography (33). Therefore, throughout the analytical process, the descriptive themes and subthemes were organized into higher-order themes by considering what described different nuances or aspects of the same overarching theme. The first author’s summary of the descriptive themes enabled reflection of the content across the descriptive themes and provided new perspectives and meaning of the content where new patterns became visible, resulting in the development of the analytical themes. The analytical themes developed in this thematic synthesis are presented in **Table 1**.

**Table 1:** Overview of analytical and descriptive themes.

Analytical themes	Cultural variability and adaptability in understanding of mental health			Help-seeking behavior among refugee youths consists of social and self-help strategies for mental health, and a limited use of healthcare services due to cultural barriers.		
Descriptive themes	Understanding of mental health and mental health problems as a dysfunction of body and mind	Understanding of mental health and mental health problems as related to spiritual or religious beliefs	Understanding of mental health and mental health problems as related to concerns about their life situation	Religious and spiritual practices to manage mental health problems	Seeking help from professionals and mental health services	Seeking help from family, friends and self for their mental health.

## 3 Results

### 3.1 Literature search results

**Figure 1:**
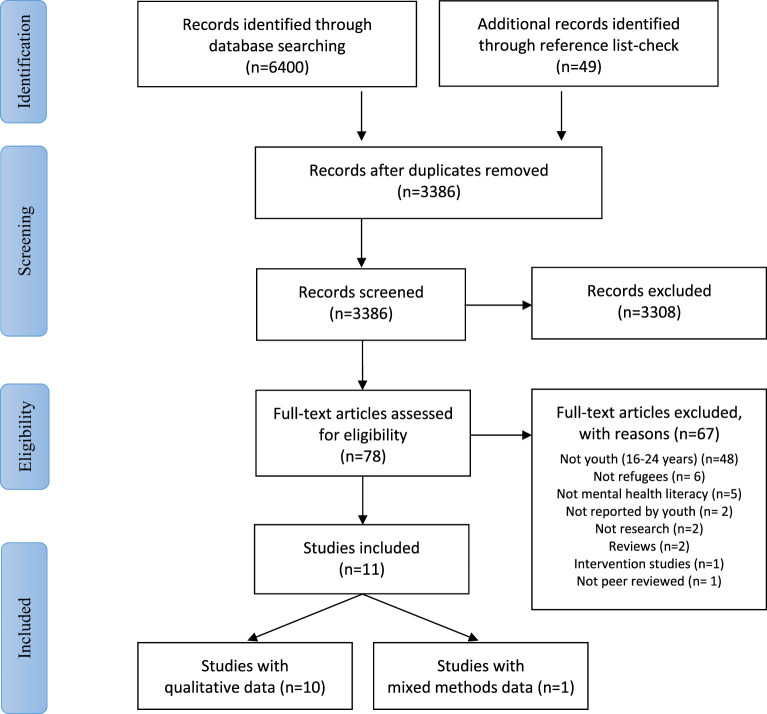
PRISMA flow diagram for the systematic review.

A total of 6,400 titles were identified through the database searches. After removal of duplicates and adding titles identified through screening of reference lists, a total of 3,386 unique titles remained. All titles and abstracts were screened to assess their potential to meet the inclusion criteria. This process led to consideration of 77 full-text articles, of which 11 were ultimately included (35–45). (For further details, see **Figure 1**).

### 3.2 Quality assessment of the included studies

The included studies provided sufficient information in the abstract, introduction, results and discussion sections. Several studies included no (35, 37–42, 44, 45) or only partial (36, 43) information about methods in the title, but this information was provided in the abstract and methods sections. The methods sections were insufficient in some articles, as they lacked information about data saturation (35, 37–40, 44, 45), the point in time of data collection (38, 40–43, 45), and measures applied to enhance trustworthiness (35, 37, 39–42). Another limitation was that conflicts of interest (35, 36, 38, 40–42, 45) or funding (35–37, 39, 41, 42, 45) were not reported for some of the studies. (More on quality assessment in **Table 2**).

**Table 2:** Results Standard for Reporting Qualitative Research (SRQR).

	First author, year, (reference)	Copolov 2023 (35)	De Anstiss 2010 (36)	Filler 2021 (37)	Finnigan 2022 (38)	Jarlby 2018 (39)	Majumder 2015 (40)	Majumder 2019 (41)	Majumder 2019 (42)	Saberi 2021 (43)	Van der Meer 2023 (44)	Wittaker 2005 (45)
Title& abstract	S1 Title	N	Y	N	N	N	N	N	N	Y	N	N
	S2 Abstract	Y	Y	Y	Y	Y	Y	Y	Y	Y	Y	Y
Introduction	S3 Problem formulation	P	P	Y	Y	P	Y	Y	Y	Y	Y	Y
	S4 Purpose or research question	Y	Y	Y	Y	Y	Y	Y	Y	Y	Y	Y
Methods	S5 Qualitative approach and research paradigm	Y	N	Y	Y	N	Y	Y	Y	Y	Y	Y
	S6 Researcher characteristics and reflexivity	N	N	N	N	N	N	Y	N	N	N	Y
	S7 Context	N	Y	N	N	Y	N	N	N	Y	Y	Y
	S8 Sampling strategy	P	Y	P	P	P	P	Y	Y	Y	P	P
	S9 Ethical issues pertaining to human subjects	Y	N	Y	U	U	Y	Y	Y	Y	Y	Y
	S10 Data collection methods	Y	P	Y	P	Y	P	N	P	P	Y	P
	S11 Data collection instruments and technologies	Y	Y	Y	Y	Y	P	N	P	Y	Y	Y
	S12 Units of study	Y	Y	Y	Y	Y	Y	Y	Y	Y	Y	Y
	S13 Data processing	P	P	N	Y	Y	P	P	N	P	Y	P
	S14 Data analysis	Y	Y	Y	Y	Y	Y	Y	Y	Y	Y	Y
	S15 Techniques to enhance trustworthiness	N	Y	N	Y	N	N	N	N	Y	Y	Y
Results/findings	S16 Synthesis and interpretation	Y	Y	Y	Y	Y	Y	Y	Y	Y	Y	Y
	S17 Links to empirical data	Y	Y	Y	Y	Y	Y	Y	Y	Y	Y	Y
Discussion	S18 Integration with prior work, implications, transferability, and contribution(s) to the field	Y	Y	Y	Y	Y	Y	Y	Y	Y	Y	Y
	S19 Limitations	Y	Y	Y	Y	Y	Y	Y	Y	Y	Y	Y
Other	S20 Conflicts of interest	N	N	Y	N	Y	N	N	N	Y	Y	N
	S21 Funding	N	N	N	Y	U	Y	N	N	Y	Y	N
	Quality score	13/21	14/21	13,5/21	14/21	13,5/21	13/21	13/21	11,5/21	18/21	17,5/21	15,5/21

Y, Yes; P, Partly Yes; N, No; U, Unclear.

### 3.3 Study characteristics

The 11 included studies used qualitative methods, with the exception of one which used mixed methods (36). However, only the qualitative data in this study fulfilled the inclusion criteria. Individual interviews alone (n=7) or in combination with focus groups (n=3) were most common, whereas one study solely used focus group interviews (36). The most common analytical method was thematic analysis.

Most (n=9) studies had been published over the past 10 years. Six studies explored how youths understand or conceptualize mental health (37, 39–41, 44, 45); three explored barriers and facilitators to service use (36, 38, 42); and two studies explored both (35, 43).

A total of 201 youths participated in the 11 studies. The same participants were used in three studies, but they were only counted once (40–42). Five studies reported mainly on the perspectives of male participants (39–42, 44), two included solely male participants (35, 40), and one only female participants (45). Several studies reported on unaccompanied minors, of which most were male (39–42, 44). Female refugee youths, on the other hand, were mostly accompanied by their families (35, 43, 45). Most participants originally came from countries or areas in the Middle East or Africa and were at the time of the study living in Europe, Canada, or Australia. The study characteristics are presented in **Table 3**.

**Table 3:** Study characteristics.

Author, year	Methods	Main aim	Country Setting	When conducted	Study population	Time spent in current country	Refugee status	Accompanied or unaccompanied
Copolov & Knowles, 2023 (35)	Qualitative study. Semi-structured interviews. Thematic analysis	To explore barriers and facilitators to care with a community sample of young Hazara refugees in Australia. Included research questions about conceptualization of mental health.	Australia, Perth, Melbourne and Sydney	Data collected between December 2015 and January 2016	18 young adults, (9 male and 9 female) aged 18-30 (Mean age 22) Hazaras with a refugee background from Afghanistan or Pakistan.	On average 7 years (range 1-16 years)	Family reunion n=9 Asylum-seekers n=8 Refugee status n=1 Self-identified as having a refugee background at the time of participation.	Males came unaccompanied. Females came with their families.
De Anstiss & Ziaian, 2010 (36)	Mixed-methods study. Extracted exclusively qualitative data: Semi-structured focus group interviews (n=13). Thematic analysis.	To explore rates and patterns of service utilization across service sectors, use of informal support, and actual and perceived barriers to service use.	Australia, not mentioned where.	Not mentioned	85 adolescents (44 male, 41 female) aged 13–17 from Afghanistan, Bosnia, Iran, Iraq, Liberia, Serbia, and Sudan.	Not stated.	Most of the adolescents arrived in as refugees or as the children of refugees. A small number of Afghan adolescents initially arrived as asylum seekers and/or family sponsored arrival.	Not stated.
Filler et al., 2021 (37)	Qualitative study. Semi-structured interviews. Grounded theory analysis approach.	To explore how Syrian refugee adolescents conceptualize mental health through their own perspectives and through service providers.	Canada, The Greater Toronto Area	Data collected between January and March 2018	7 Syrian adolescents, aged 16-19, and 8 service providers.	< 5 years	Not stated.	Not stated.
Finnigan et al., 2022 (38),	Qualitative study. Individual interviews. Group concept mapping analysis strategy	Identify barriers to accessing mental health services by migrant youth in a middle-sized central Canadian city.	Canada. Middle-sized central Canadian city.	Not stated.	30 migrants aged 16-22 (mean age 18) from Syria, Iraq, Eritrea, Columbia, Afghanistan, Pakistan, Austria, Congo, Palestine and Ethiopia.	Mean and median number of months the youth lived in Canada was 30 (mode=36).	Not stated	Not stated.
Jarlby et al., 2018 (39)	Qualitative study. Participant observation, semi-structured individual interviews and a focus group interview.	To explore how unaccompanied refugee adolescents perceive mental health and the mental healthcare, and which services they believe would benefit them.	Denmark. In a municipal institution for young refugees in rural Denmark.	April 2017	6 male adolescents refugee aged 17-18, of whom three originated in the Middle East and three originated in Southeast Asia.	Length of stay in Denmark was 2–3 years (including the time as asylum seekers), while their length of stay in the municipality was on average 1.5 years.	Temporary residence permits from 1 to 5 years	Unaccompained
Majumder et al., 2015 (40)	Qualitative study. Semi-structured interviews. Thematic analysis.	To explore unaccompanied minors’ perceptions about mental health and services.	England.	Not stated.	15 adolescents (14 male, 1 female) aged 15-18 from either Arab or East African countries, and their carers.	Not stated.	Asylum-seeking (n=8) Refugee (n=7).	Unaccompained
Majumder, 2019 (41)	Qualitative study, Semi-structured interviews. Thematic analysis.	1. To explore the beliefs and perceptions of unaccompanied refugee children about mental illness, its treatment, service engagement and the stigma attached to it. 2. Develop a better understanding of the possible mitigating factors to facilitate engagement with services.	United Kingdom, Central part.	Not stated.	15 adolescents (14 male, 1 female) aged 15-18 from either Arab or East African countries, and their carers.	Not stated.	Asylum-seeking (n=8) Refugee (n=7)	Unaccompained
Majumder et al, 2019 (42)	Qualitative study. Semi-structured interviews. Thematic analysis.	To explore mental health services from the perspective of unaccompanied refugee minors and their carers, to understand the possible barriers to engagement and to identify aspects of service provision that contribute to the observed poor service access and treatment engagement.	England.	Not stated.	15 adolescents (14 male, 1 female) aged 15-18 from either Arab or East African countries, and their carers.	Not stated.	Asylum-seeking (n=8) Refugee (n=)	Unaccompanied
Saberi et al., 2021 (43)	Qualitative study. Individual and focus group interviews. Inductive thematic analysis.	To explore understandings of young Hazara refugees in Melbourne of mental health issues and barriers to accessing primary mental health care.	Australia, Melbourne.	2014	17 Hazaras aged 18–30 years.	Between 1 to 5 years	The male participants were asylum-seekers. Female participants had permanent visas.	Males came unaccompanied. Females came with their families.
Van der Meer et al., 2023 (44)	Qualitative study. Individual interviews. Content analysis.	To analyze the conceptions of (mental) health and (mental) illness of refugee children, adolescents, and young adults in Germany.	Germany.	April 2019 to October 2020.	18 male respondents aged 10 - 21 from the Middle East and East Africa.	Not stated.	Not stated.	Unaccompanied.
Wittaker et al., 2005 (45)	Qualitative study. Semi-structured individual and focus group interviews. Interpretative Phenomenological Analysis.	To explore the individual and interpersonal or collective perceptions of psychological well-being among young Somali female asylum-seekers and refugees, growing-up in northern England	United Kingdom. Northern England.	Not stated.	5 female aged 17-28, from Somalia.	From 1-12 years.	Asylum-seekers (n= 2) Refugees (n=2) Family reunion (n=1)	Accompanied

### 3.4 Analytical and descriptive themes

Two analytical themes were developed to describe the existing research literature reporting on MHL among refugee youths: *Cultural variability and adaptability in understanding of mental health*; and *Help-seeking behavior among refugee youths consists of social and self-help strategies for mental health, and a limited use of healthcare services due to cultural barriers.* Although there were some similarities across the themes, they described distinctly different aspects.

#### 3.4.1 Cultural variability and adaptability in understanding of mental health

The first analytical theme was derived from three descriptive themes: *Understanding of mental health and mental health problems as a dysfunction of body and mind*; *Understanding of mental health and mental health problems as related to* sp*iritual or religious beliefs;* and *Understanding of mental health and mental health problems as related to concerns about their life situation*.

This analytical theme illustrates how the understanding of mental health and mental health problems can vary among young refugees. Such problems were described as connected to body and mind; related to religious and spiritual beliefs; to concerns about their life situation; or lack of awareness about mental health altogether. However, the youths’ understandings changed over time, particularly for those who associated mental health with religious or spiritual beliefs. Changes occurred for some after having lived in a new country for a longer period of time. Relocating to another country contributed to gaining new knowledge and for youths to distance themselves somewhat from previous beliefs. Several youths believed that the causes of mental health problems were related to post-migration stressors such as being a refugee, concerns for their families, a state of solitude, feeling uncertain about the future and the economic situation, and the uncertainties associated with their visa status. They were more concerned with their current situation and future prospects, and actively working to improve their life situation, rather than dwelling on the past.

##### 3.4.1.1 Understanding of mental health and mental health problems as a dysfunction of body and mind

Several youths related mental health and mental health problems to the function or dysfunction of body and mind. This was manifested as a belief about the interconnection between body and mind, understanding mental health as connected to physical health (35), or to the mind (44). Mental health and mental health problems were described in various ways. Some youths referred to mental health problems as not being happy (40); and mental health was referred to as not having excessive worries (44). Others used diagnostic terms such as anxiety and depression to explain mental health (44). Stigmatizing terms were used by several participants, such as being “crazy” (36, 37, 40, 41, 43) “weird”, “psycho” (36, 43) “mad” (41), or “sick in the head” (44). Youths feared mental disorders due to the stigma of being labelled as “crazy” (37, 41) and fear of being locked up in hospitals or prisons (41). However, more commonly less stigmatizing terms were used to describe mental health problems, such as stress, pressure and discomfort (37). For some youths, the perception of mental health remained unchanged over time (41, 44), even after contact with the mental health services in the new country (41).

Mental health was also perceived by some to be connected to cognitive functioning and the presence or absence of illness such as Alzheimer’s disease (37). Yet others described mental health as manifested by somatic complaints or bodily symptoms (35, 37, 40, 41), or not taking care of their hygiene or appearance (37). While some of the youths recognized mental health problems as health problems, they reported that older members of the community rather thought about it as a natural process (35). Other youths did not know what mental illness was, and some did not know how to explain it (37, 44).

##### 3.4.1.2 Understanding of mental health and mental health problems as related to spiritual or religious beliefs

Due to influences of spirituality or religion, some perceived mental health as an external “force of nature” that could occupy the body and affect the mind (44). Both mental and somatic symptoms could be described as a result of possession by spirits, and illness was understood as malediction (45). These beliefs seemed to be more common among recently arrived refugee youths (45). For some, their understanding of mental illness changed after having lived in a new country for some time, including their knowledge about the potential causes of mental health, differences between mental and somatic illness, and interventions that could be used to treat or prevent mental illness. They changed their perspectives from one focusing on religion and spirits, to illness and prevention of mental health (44). Over time, youths became more skeptic of traditional beliefs, such as “jinn” and “zār” spirits (45). “Jinn” spirits are grounded in the Islamic teaching in the Quran, and “zār” spirits originate from African traditions (45). They considered such beliefs as a way for the elderly in their community to hold on to their culture. Within the context of their new country, the youths felt no need to hold on to such beliefs (45).

##### 3.4.1.3 Understanding of mental health and mental health problems as related to concerns about their life situation

Different stressors seemed to be perceived as important reasons for mental health problems. Stress related to being a refugee was mentioned by several unaccompanied male refugees (35, 39, 40, 43). Such stress was perceived both at an individual level with the experiences of trauma, forced migration and residing in detention centers; and at the collective level, as having a sense of being abandoned by the rest of the world, and a sense of injustice imposed upon them by the majority population community (43). Poor mental health was also understood as caused by loneliness (35, 39), which could occur upon arriving in a new country without family support (35). Uncertainty about the future, particularly regarding permission to stay in the destination country (43), feeling judged as lower status citizens (43), and economic worries (36, 39) were additional factors. Concerns about family members also contributed to mental distress (35, 40). Moreover, discrimination due to being treated differently from the majority population resulted in feelings of inferiority (43). Exposure to racism and harassment was another cause of mental health problems (36). Factors promoting good mental health included participating in meaningful activities, being physically active, taking on responsibilities, and attending work or school (37, 39, 45). Moreover, promoting equal rights for boys and girls was seen as supporting equity in life opportunities, such as in the workplace, and encouraging a focus on the future rather than the past, which could support mental health. Mental health was also associated with strong social relationships and support, functioning well in daily life, and experiencing the happiness of friends and family (39). After fleeing from their country of origin and gaining new knowledge, some realized that enjoyable activities could positively influence their mental health (44).

#### 3.4.2 Help-seeking behavior among refugee youths consists of social and self-help strategies for mental health, and a limited use of healthcare services due to cultural barriers

The second analytical theme; Help-seeking behavior among refugee youths consists of social and self-help strategies for mental health, and a limited use of healthcare services due to cultural barriers; was derived from three different descriptive themes: Religious and spiritual practices to manage mental health problems; Seeking help from professionals and mental health services; and Seeking help from family, friends and self for their mental health.

Refugee youths sought help from various sources, depending on what they perceived to be helpful. Several youths turned to religious and spiritual practices to manage mental health problems, whereas talking about mental health problems with either professionals or others, was often not perceived to be helpful. Reasons for this included fear of aggravating the problem, stigma, and concerns about not being understood, judged or ignored. There were also worries about confidentiality from professionals, friends, and others. Trust in professionals and others was a significant factor in the willingness to seek help, with trust being more important than cultural similarity. There was a discrepancy in the understanding between what refugee youths needed and what professionals offered; youths wanted to discuss their present and future, whereas professionals focused on their past.

##### 3.4.2.1 Religious and spiritual practices to manage mental health problems

Several of the youths talked about different religious and spiritual practices as a way of seeking relief from mental distress and help for mental health problems. This ranged from reading the Quran, praying, seeking help from an Imam or a priest (44, 45), practicing different religious or spiritual rituals (35, 36, 39, 43–45), or using herbal medicine (36, 39). For some youths this was related to the belief that prayers, rituals or reading the Quran could protect or release them from spiritual possession (45). Others believed that mental illness was cured or prevented by such practices (36, 44). Females were more likely than male participants to use religious practices to manage psychological distress (35).

##### 3.4.2.2 Seeking help from professionals and mental health services

There was considerable variation in refugee youths’ awareness of the existing mental health services and their beliefs about the helpfulness of such services. Some thought the existing services were only there for the majority population of youths (45). Others believed that the services could be helpful, but they were too afraid of being sanctioned by their families to make use of them (44). Such services were not frequently used in their country of origin due to lack of financial resources needed to enable them to access the services (40), and basic needs such as food and housing were prioritized (44). Some thought medication could be helpful (42), whereas others were concerned about the risk of becoming addicted to them (35). Psychotherapy was considered to be helpful by some (37, 42, 44), whereas others did not find such therapies helpful (36, 38, 45). Negative beliefs or experiences discouraged youths from sharing their problems with professionals (35, 36, 38–43, 45). Talking therapies could bring back bad memories from the past and aggravate their mental health (39, 42, 43). These youths preferred to talk about their current challenges, rather than focusing on the past (42). Furthermore, they felt that healthcare professionals often lacked cultural competence, resulting in youths not feeling understood (36, 38, 40, 45). This could lead to drop-out from treatment (43).

Youths had different opinions as to whether professionals should have the same cultural background as themselves. Cultural background was by some not considered important, as long as there were no language barriers (37). This hindered their ability to express themselves about mental health problems and to access services (40, 43). On the other hand, cultural background was important for some in order to be able to trust professionals. They avoided seeking help from professionals with the same cultural background due to concerns that information might be shared with their community or parents (36). Others felt unsafe discussing their problems with professionals of a different ethnic background, fearing breaches of confidentiality that could result in their parents learning about their mental health problems (38, 40). Healthcare professionals were perceived to be very busy, and the youths did not believe their problems were severe enough to warrant professionals’ time. Moreover, youths were afraid of being misunderstood, ignored or judged (38). The gender of professionals was another aspect that was brought up by some of the youths. Male professionals were perceived to be better able to carry the burden of the youths’ problems than female professionals, and consequently male patients could be reluctant to talk to female therapists. For others, however, it was more important that the health professional had a sense of humor and showed interest to talk with them (42).

##### 3.4.2.3 Seeking help from family, friends and self for their mental health

Youths were likely to seek help from family and friends, or to manage mental health problems on their own. They could talk about their problems with people they knew, although some kept personal information to themselves. Family was seen as an important source of support (37, 45). Their mothers in particular played an important role in taking care of the entire family’s well-being (45). The perception of the family being supportive seemed to be most common in those who related their perceptions of mental health to religious and spiritual causes. On the other hand, some youths would not share their mental health problems with their parents. Firstly, they feared their parents’ reactions, and potential sanctions (43, 45). Secondly, mental health problems were considered to be unacceptable by some parents who projected guilt and shame onto their youths if they discussed mental health problems outside the community (35, 36, 38), as mental health was considered taboo (36, 38), or a shameful topic that should remain private and kept within the family (36). Others feared that the authorities might learn about their problems, which could endanger the situation for their family (38), or affect their visa status (35). Thirdly, a lack of emotional attachment between parents and youths prevented them from sharing their thoughts and feelings (36). According to these youths, parents lacked knowledge about mental health and would not understand (36, 43), might worry (35), or be unable to carry the burden and provide positive support (36). Education and information about mental health for mothers was mentioned by some participants as a means to improve access to mental health services (43).

Other youths preferred to seek help or advice from friends rather than parents or family members (36, 45), because of the inter-generational differences in experiences, values and behavioral norms (36). Friends from different ethnic backgrounds seemed important to some youths, while others chose not to tell anyone about their mental health problems due to a lack of trust in anyone and fear of gossip and judgement (36, 38, 45). Some of the youths believed no one could help but themselves, and various self-help strategies were applied, ranging from exercising and writing down thoughts to deal with mental stress, whereas others used substances to deal with distress (35).

## 4 Discussion

This is the first systematic review reporting on MHL in youths with a background as refugees. The participants in the included studies described different understandings of mental health and mental health problems. For some, it was connected to body and mind; for others, it was tied to religious and spiritual beliefs, or concerns about their life situation. Yet others, were unable to understand or to explain the concepts of mental health and mental health problems. Consequently, this resulted in different ways of managing mental health problems. Some sought relief through spiritual and religious practices, others found support in family and friends, whereas yet others sought help from mental health professionals. Many youths were aware of the existing mental health services, but barriers prevented them from seeking professional help, such as lack of cultural competency among professionals and concerns about mental health stigma.

Within the Western healthcare services, there is a predetermined view that youths should possess a certain degree of MHL to utilize available services (22, 24). Such a perspective may place an expectation on refugee youths to adopt a Western understanding of mental health to access the services. Consequently, MHL might be seen as a barrier to professional help-seeking among refugee youths. We argue that this view maintains inequity in mental health provision and unequal opportunities for achieving good mental health. Hence, it may not be the MHL of refugee youths that serves as a barrier to service use, but rather the current definition and interpretation of the meaning and role of MHL.

### 4.1 The relevance of mental health services to meet youths’ needs

This review reveals a gap between the factors youths identify as causing mental health issues and the support provided by mental health services and professionals. It underscores the importance of cultural competency, trust, and addressing stigma. This gap creates a barrier to accessing mental health services and developing effective MHL programs. To reach refugee youths effectively and provide tailored support, service providers must understand service users, their perspectives on mental health, and how to build trust to meet their needs.

Several youths considered talking about their problems with professionals to be unhelpful, and for some it resulted in dropout from treatment (35, 36, 38, 39, 41–43, 45). This could be interpret as having a low MHL and it could be explained as reluctance to seek professional help. However, from the perspective of youths, the services provided for them did not meet their needs (42, 45). One reason for this was that lack of cultural competency among professionals which served as a barrier to seek professional help (36, 38, 40, 45). These findings are supported by Colucci et al. (46), who found that healthcare professionals bring up trauma too early, they do not acknowledge the impact of resettlement and the different ways in which mental health may be understood by youth. Moreover, they ignore and underestimate the role of family and community. To facilitate service access, it is important to recognize that youths’ priorities might differ from those of professionals, as well as to engage families and the community. For example, the majority of Asian cultures emphasize the importance of the community (45), where families and local networks play a crucial role in providing care and support to individuals, in contrast to more individualistic cultures. However, it is essential to take into account the preferences of refugee youths, as some may prefer not to disclose their mental health issues to their families or communities (35, 36, 43).

In Western cultures, mental health is often understood as a continuum from poor to good, and it is considered an integral part of mental well-being (47). Such a view makes it easier to talk about mental health as a normal part of life. Refugee youths may associate mental health with diagnoses and serious conditions, and they express their concerns by using terms such as “crazy”, “mad”, and “locked up”. This implies that it pertains only to serious mental disorders, and therefore, to a small proportion of the population. Hence, the differing perspectives result in a lack of mutual understanding. The disparity in understanding mental health between refugee youths and professionals creates a barrier to accessing services.

Different ways of understanding mental health lead to different beliefs about how to manage psychological distress. Western treatment approaches to trauma often involve confronting traumatic memories and discussing the emotions that arise, while this practice is less common in non-Western cultures (48). Moreover, the consequences of trauma exposure can also result in retraumatization (49). This is in line with the experiences expressed by youths in this review, as they found that talking about past traumas simply made things worse (39, 42, 43). Instead, they expressed a need to manage their current challenges and focus on their future (37, 39, 42, 45).

### 4.2 Mental health literacy definition as a barrier

The concept of MHL, originally defined from a Western biomedical perspective (22), lacks a universally accepted explanatory model. While recent definitions acknowledge culture, age, and contextual influences (24), MHL still largely reflects Western views on mental health. As a result, individuals with different understandings might be perceived as having low MHL, reinforcing Western dominance in mental health perspectives – a legacy rooted in colonial history. Millner et al. (50) advocate for decentering Western mental health narratives and incorporating perspectives, such as collectivistic values, religion and spirituality and a focus on recovery, resilience and resistance (50). The World Health Organization and the Office of the High Commissioner for Human Rights call for a paradigm shift towards a human rights model focusing on person-centered, community-based and recovery-based practices (51, p 90). In line with this, the current review highlights the need for a broader understanding of mental health that acknowledges cultural and contextual differences in how mental health is understood, managed, and promoted.

Understandings of mental health, mental health problems, and the need for mental health support are influenced by both culture and context. MHL encompasses both the awareness and knowledge of mental health among service users, as well as the knowledge and competence among professionals working within the services. The definition of MHL originates from the concept of health literacy (19, 24). Over the past decade, the term organizational health literacy has been defined as *“the degree to which organizations equitably enable individuals to find, understand, and use information and services to inform health-related decisions and actions for themselves and others”* (52). Addressing MHL within services aims to enable individuals to access and use information and decrease the demand of MHL in the individuals (53, 54). It has been proposed to expand the definition of health literacy to include an organizational component and to improve health literacy awareness among health professionals and organizations (54). It is necessary to increase professionals’ knowledge about youths’ understanding of mental health, mental health problems, beliefs about causes, help-seeking behaviors, as well as to agree on and use terms that are acceptable and relatable among refugee youths. However, the term mental health literacy could be questioned, given that mental health is complex and shaped by culture and context. There is no single approach to preventing mental health issues or promoting well-being, and there are diverse sources for seeking help.

### 4.3 User perspectives to improve knowledge about youths’ mental health needs

The right to be heard is given in Article 12 of the United Nations Convention on the Rights of the Child (55). Youths’ understanding of mental health and mental health problems, and ways to prevent, manage and promote overall well-being, must be explored, rather than measuring levels of mental health literacy. Professionals often lack knowledge about the self-defined mental health needs of refugee youths. Peer involvement and support from persons with lived experiences are offered as options to provide culturally sensitive services (51). A review of user involvement in adolescents’ mental healthcare revealed that adolescents want to be heard and involved in decisions affecting their mental health at both an individual and an organizational level (56). Their involvement can contribute to strengthen the relevance, appropriateness and acceptability of treatment, and to influence treatment programs and interventions (56). Moreover, focus on shared decision-making and person-centered care in professionals’ training, can contribute to empower adolescents to take more responsibility and action in their own recovery processes (56). A previous study found that healthcare professionals propose measures to strengthen user involvement through means such as training for healthcare professionals, changes in workplace culture and flexible approaches adapted to the users’ needs (57). Although there are examples of research focusing on co-creation in mental health services (58, 59), there are still barriers to implement co-creation in practice (60). Different conceptualization of co-creation leads to different understanding of the concept and how co-creation is implemented into practices. Building trusting relationships between professionals and refugee youths is challenging, and a lack of cultural understanding further complicates the opportunity of co-creation (60). Consequently, the rights of refugee youths to be involved in decision making concerning their mental health and in adaption of mental health services continue to be neglected.

### 4.4 Strengths and limitations

This is the first review which explores MHL among refugee youths. Their perspectives are crucial for assessing and further developing the concept of MHL. However, there are some limitations that are addressed below.

First, only English search terms were used. However, 104 non-English language articles were identified, but none fulfilled the inclusion criteria. It can nevertheless not be ruled out that additional articles might have been identified if literature searches had included search terms in other languages. Second, the age limitation was set from 16 to 24 years. This may have resulted in loss of potentially relevant articles focusing on youths who were younger or older (61). Third, the definition of MHL may have resulted in loss of articles and data material from the included articles. However, the aim of this review was to explore MHL.

The credibility of this systematic review was strengthened by the use of pre-defined research strategies, which were also published in PROSPERO. This included a clear description of the methods used for data collection and analysis, to ensure transparency throughout the review process. The review was conducted by four authors who have diverse professional backgrounds, collectively contributing to the analysis of data and interpretation of results. Another strength was the use of the PRIMSA statement guidelines to report the results of the literature searches. Moreover, findings are presented using examples from the included studies, thereby reflecting the voices of refugee youths.

### 4.5 Implications

This review has important implications for the concept of MHL, including the need to incorporate refugee youths’ perspectives into policies, mental health services, professional training, as well as in further research.

A significant barrier to engagement in mental health among refugee youths is the lack of cultural competency among professionals, compounded by the stigma associated with mental health problems, and the perception that discussing mental health problems might aggravate their mental health. This may create a substantial gap in understandings between individuals, professional and mental health services and negatively impact patient safety. To address this, enhanced cultural sensitivity within mental health services and increased MHL among refugee youths may help them understand mental health in a way that better aligns with their experiences and beliefs. This can be achieved by incorporating youths’ perspectives into policy making, promoting interdisciplinary services in environments where youths are present daily, such as schools and recreational settings, and prioritizing early intervention and prevention to address mental health concerns before they escalate, thereby reducing the need for more specialized services.

MHL should reflect a holistic and inclusive understanding of mental health, encompassing diverse cultural perspectives, help-seeking behaviors, self-help strategies, and social and contextual factors, rather than being solely based on a Western model. This requires a further development of MHL programs and services that include the voices of youths and incorporate cultural, age, and gender factors. Psychoeducation to reduce stigma should also be integrated into mental health literacy programs, using culturally appropriate terminology that resonates within communities which include refugees. This should be embedded into educational settings, community mental health literacy programs, and in professional development as well as in policy making to ensure a comprehensive understanding of mental health and mental health needs. The inclusion of refugee youths’ perspective is not optional, but a fundamental human right.

The studies included in this review were heterogeneous, with variations in numbers of participant, countries of origin, and whether the youths were accompanied or unaccompanied. The literature provides limited insight into the experiences and perspectives of female youths. There is limited research-based knowledge about MHL among these youths. Therefore, a variety of research both quantitative and qualitative to is needed. Further research is necessary to advance the concept of MHL and incorporate the perspectives, culture and context of youths. A tool should be developed to map MHL in accordance with these new conceptual understandings. Moreover, it is important to explore cultural competence among professionals and develop training programs that integrate the perspective of youths.

### 4.6 Conclusions

This systematic review examined the existing research literature exploring mental health literacy among refugee youths and discussed the role of mental health literacy as a barrier to help seeking. There are cultural differences in how mental health is understood among refugee youths and how mental health problems should be managed. Stigma associated with mental health continues to persist among refugee youths. However, we suggest that the main barrier to service use might not be low levels of MHL among refugee youths, but rather how mental health literacy is conceptualized, and how mental health services and professionals fail to meet youths self-defined mental health needs. Further research is needed to explore how the concept of mental health literacy can incorporate diverse perspectives on mental health. This research should aim to refine the MHL framework to encompass various cultural understandings of mental health, different approaches to managing mental health problems, and strategies for promoting good mental health.
