# Supplementary material for: Exploring mental health literacy among youths with background as asylum-seekers and refugees: a systematic review
Source: Front Psychiatry. 2025 Apr 8;16:1538946. doi: 10.3389/fpsyt.2025.1538946 (PMC12011742; doi:10.3389/fpsyt.2025.1538946)
Supplement: Supplementary file 1 [file Table1.docx]

Supplemental file 1. The systematic search and outcomes

| **Database** | **Date** | **Terms** | **Hits** | **Included articles** |
| --- | --- | --- | --- | --- |
| **EMBASE (Ovid)** | 20.12.2023 | S1. youth* OR young* OR young adult (SH) OR young adult* OR adolescent (SH) OR adolescen* OR teen*. | 3165422 |  |
|  |  | S2. Refugee (SH) OR refugee* OR asylum* OR asylum-seeker* OR exile OR fugitive* OR escapee* | 26419 |  |
|  |  | S3. mental health literacy OR mental health knowledge OR  mental health belie* OR mental health awareness OR attitude to mental health OR mental health stigma OR mental health help seeking behavior OR mental health (SH) OR mental health | 438367 |  |
|  |  | **S1 AND S2 AND S3** | **1379** | (31,32,35–39,41) |
| **CINAHL**  **EBSCO host** | 20.12.23 | S1. Young Adult (SH) OR young adults OR Young OR young people OR  youth OR Adolescence (SH) OR adolescents OR young people OR teen | 934652 |  |
|  |  | S2. Refugees (SH) OR refugee* OR asylum-seeker* OR asylum* OR exile OR  fugitive* | 12 516 |  |
|  |  | S3. mental health literacy OR mental health knowledge OR mental health belie* OR Attitude to Mental Illness (SH) OR attitudes towards mental illness OR attitudes to mental health OR mental illness stigma OR mental health stigma* OR mental health  awareness OR mental health help-seeking behavior OR Mental Health (SH) OR mental health | 192905 |  |
|  |  | **S1 AND S2 AND S3** | **760** | (31,32,35,36,38,39,41) |
| **PsycInfo (Ovid)** | 21.12.2023 | S1. Youth* OR Adolescent (SH) OR adolescent OR Young Adult (SH) OR young adult* OR  teen* OR young OR young people | 3187837 |  |
|  |  | S2. Refugees (SH) refugee* OR asylum-seeker* OR asylum* OR exile OR fugitive* | 23089 |  |
|  |  | S3. mental health literacy OR mental health knowledge OR mental health belie*  OR mental health awareness OR attitudes towards mental illness OR attitudes to mental health OR mental health stigma OR mental illness stigma OR  mental health help-seeking behavior OR mental health (SH) OR mental health | 281203 |  |
|  |  | **S1 AND S2 AND S3** | **1204** | (31–33,36) |
| **Web of Science** | 22.12.2023 | S1. youth* OR young* OR adolescent* OR teen* OR young adult* OR young people | 1644796 |  |
|  |  | S2. refugee* OR asylum* OR asylum-seeker* OR exile OR fugitive* OR escapee* OR “displaced people" | 63 976 |  |
|  |  | S3. mental health literacy OR mental health knowledge OR mental health belie* OR attitude to mental health OR mental health stigma* OR mental illness stigma OR attitudes to mental illness OR attitudes to mental health OR mental health help-seeking behavior OR health literacy OR mental health | 333107 |  |
|  |  | **S1 AND S2 AND S3** | **1671** | (31–36,38–40) |
| **MEDLINE** | 05.01.2024 | S1 youth* OR Adolescent (SH) OR adolescent* OR Young Adult (SH) OR young adult* OR teen* OR young OR young people | 3190033 |  |
|  |  | S2. Refugees (SH) OR refugee* OR asylum-seeker* OR asylum* OR exile OR fugitive* | 23135 |  |
|  |  | S3. mental health literacy OR mental health knowledge OR mental health belie* mental health awareness OR mental health stigma* OR mental illness stigma OR attitudes to mental illness OR attitudes to mental health OR mental health help-seeking behavior OR mental health (SH) OR mental health | 282041 |  |
|  |  | **S1 AND S2 AND S3** | **1211** | (31,32,35,39,40) |
| **PubMed** | 02.01.2024 | Adolescent (SH) OR adolescent OR young adult (SH) OR young adult OR teen* OR young people OR young | 3264651 |  |
|  |  | Refugees (SH) OR refugee OR asylum-seeker* OR fugitive* OR exile OR escape* | 18933 |  |
|  |  | mental health literacy OR mental health knowledge OR mental health awareness OR mental health stigma OR mental illness stigma OR mental health belie* OR attitudes towards mental health OR attitudes towards mental illness OR mental health help-seeking behavior OR mental health (SH) OR mental health | 306 825 |  |
|  |  | S1 AND S2 AND S3 | 1078 |  |
|  |  | **S1 AND S2 AND S3. Filters from 2022-2024** | **175** | (31,40) |
| **Total** |  |  | **6400** |  |

SH= Subject heading (Medline and CINAHL: MeSH terms, Embase: emtree – a list of subject headings unique to Embase).
